# Predicting central lymph node metastasis in papillary thyroid microcarcinoma: a breakthrough with interpretable machine learning

**DOI:** 10.3389/fendo.2025.1537386

**Published:** 2025-05-12

**Authors:** Weijun Zhou, Lijuan Li, Xiaowen Hao, Lanying Wu, Lifu Liu, Binyu Zheng, Yangzheng Xia, Yong Liu

**Affiliations:** Department of Ultrasound, Beijing Shijitan Hospital, Capital Medical University, Beijing, China

**Keywords:** machine learning, papillary thyroid microcarcinoma, central lymph node metastasis, diagnostic imaging, SHapley Additive exPlanation

## Abstract

**Objective:**

To develop and validate an interpretable machine learning (ML) model for the preoperative prediction of central lymph node metastasis (CLNM) in papillary thyroid microcarcinoma (PTMC).

**Methods:**

From December 2016 to December 2023, we retrospectively analyzed 710 PTMC patients who underwent thyroidectomies. Feature selection was conducted using the least absolute shrinkage and selection operator (LASSO) regression method, alongside the Support Vector Machine-Recursive Feature Elimination (SVM-RFE) algorithm in conjunction with multivariate logistic regression. Eight ML algorithms, namely Decision Tree, Random Forest (RF), K-nearest neighbors, Support vector machine, Extreme Gradient Boosting, Naive Bayes, Logistic regression, and Light Gradient Boosting machine, were developed for the prediction of CLNM. The performance of these models was evaluated using area under the receiver operating characteristic curve (AUC), decision curve analysis (DCA), sensitivity, specificity, accuracy, positive predictive value (PPV), negative predictive value (NPV), and F1 scores. Additionally, the Shapley Additive Explanation (SHAP) algorithm was utilized to clarify the results of the optimal ML model.

**Results:**

The results indicated that 32.95% of the patients (234/710) presented with CLNM. Tumor diameter, multifocality, lymph nodes identified via ultrasound (US-LN), and extrathyroidal extension (ETE) were identified as independent predictors of CLNM. The RF model achieved the highest performance in the validation set with an AUC of 0.893(95%CI: 0.846-0.940), accuracy of 0.832, sensitivity of 0.764, specificity of 0.866, PPV of 0.743, NPV of 0.879, and F1-score of 0.753. Furthermore, the DCA demonstrated that the RF model exhibited a superior clinical net benefit.

**Conclusion:**

Our model predicted the risk of CLNM in PTMC patients with high accuracy preoperatively.

## Highlights

- This is a retrospective study to analyze the possibility of the machine learning (ML) model preoperatively predicting Cervical lymph node metastases (CLNM) in Papillary thyroid microcarcinoma (PTMC).
- The model established by combining US image features and clinical features has high predictive performance in both derivation cohort and external validation cohort.
- The findings can help clinicians predict CLNM before surgery and provide a basis for monitoring strategies for PTMC patients.

## Introduction

Papillary thyroid carcinoma (PTC) represents the most prevalent histological subtype of thyroid malignancy, comprising over 80%~90% of all thyroid carcinoma cases (1, 2). The incidence of papillary thyroid carcinoma (PTC) has risen, driven largely by increased detection of papillary thyroid microcarcinoma (PTMC; ≤10 mm) (3). It is believed that PTMC is a relatively indolent carcinoma, typically occurring incidentally and occult, with a good prognosis and a favorable outcome (4–6). According to the 2015 American Thyroid Association (ATA) guidelines, active surveillance is more appropriate for patient with low-risk PTMC (7). However, certain cancer cells have the potential to metastasize to the lymph nodes surrounding the thyroid gland, with a particular propensity for the cervical lymph node. Cervical lymph node metastases (CLNM) have been documented to occur in 12.3% to 49.1% of patients with PTMC (8, 9). Moreover, PTMC patients with both central and lateral nodal metastases showed a significantly lower survival rate than those who didn’t have lymph nodes involved and prone to local recurrence or distant metastasis (8).

In patients with PTMC exhibiting CLNM, central lymph node dissection (CLND) holds significant clinical importance in the management of the disease. Therefore, it is very necessary to evaluate CLNM in thyroid cancer patients before surgery. The accurate identification of CLNM preoperatively and noninvasively can improve treatment planning and eliminate unnecessary surgical intervention. The Japanese Consensus Statement on managing low-risk PTMC recommends using ultrasound to check for extra-thyroid invasion and cervical lymph node metastasis (10). However, ultrasound is highly specific for diagnosing cervical lymph nodes but less sensitive for paratracheal and retropharyngeal nodes in the central region (11). Its accuracy for detecting CLNM is also affected by inter-operator variability, highlighting the need to improve preoperative prediction precision. This may result in an inability of the required thermal ablation therapy effect and leading to higher rates of recurrence. To improve the accuracy of CLNM evaluation in thyroid cancer, many scholars analyzed the ultrasonic morphological features of thyroid cancer. Studies revealed that ultrasonic features such as microcalcification, extrathyroidal extension (ETE), ill-defined margin and internal heterogeneous low-enhancement were significant independent predictors for CLNM (12, 13).

Recently, there has been growing interest in applying machine learning (ML) for lymph node metastasis prediction from cancer imaging data. However, preoperative prediction of lymph node metastasis is challenging. Compared to other statistical models, ML makes no assumptions. Furthermore, ML has demonstrated utility in predicting lymph node metastasis in patients with thyroid cancer, with certain ML models exhibiting high predictive accuracy (9). As we know, besides accuracy, interpretability is also vital in ML. Regrettably, the majority of algorithms lack transparency, rendering the relationship between variables and outcomes indiscernible to users. Consequently, most predictive models lack interpretability in identifying high-risk features. To implement model interpretability, the Shapley Additive Explanation (SHAP) method was proposed, which is based on game theory and has been applied to tree-based algorithms to understand the predictions based on the model. In this paper, we developed and validated an interpretable ML model to predict cervical CLMN risk in PTMC and to highlight the most important sequence features.

## Materials and methods

### Participants

Data from 2450 patients who had lobectomy or total thyroidectomy with CLND between December 2016 and December 2023 at Beijing Shijitan Hospital were retrospectively collected. This retrospective study received approval from the Institutional Review Board of Beijing Shijitan Hospital (IIT2024-078-001), with a waiver of informed consent granted due to its retrospective design. The scope of thyroid surgery adhered to the ATA management guidelines (7).

**Figure 1:**
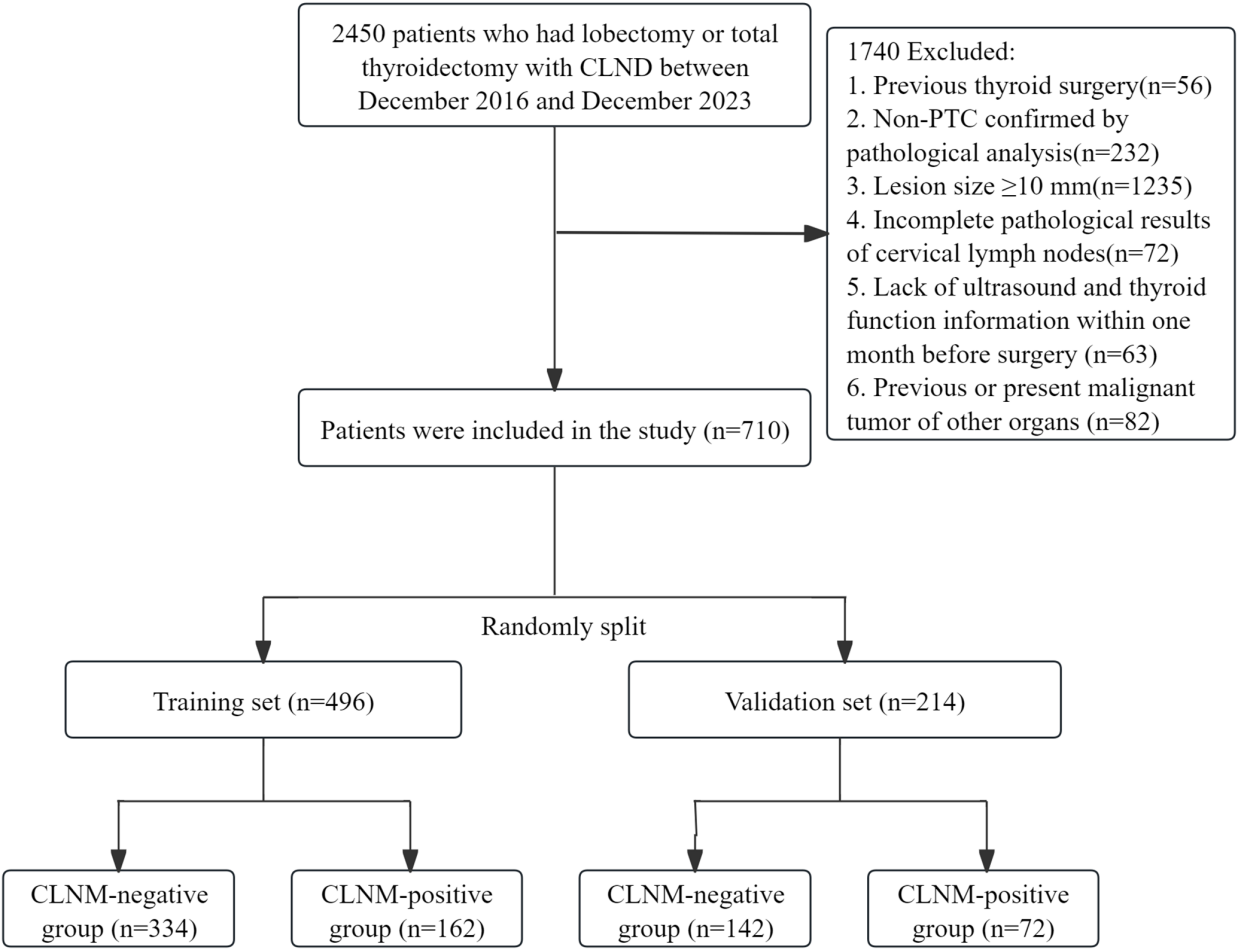
Patient flowchart for this study. CLND, central lymph node dissection; PTC, papillary thyroid carcinoma; PTMC, papillary thyroid microcarcinoma; CLNM, central lymph node metastasis.

**Figure 2:**
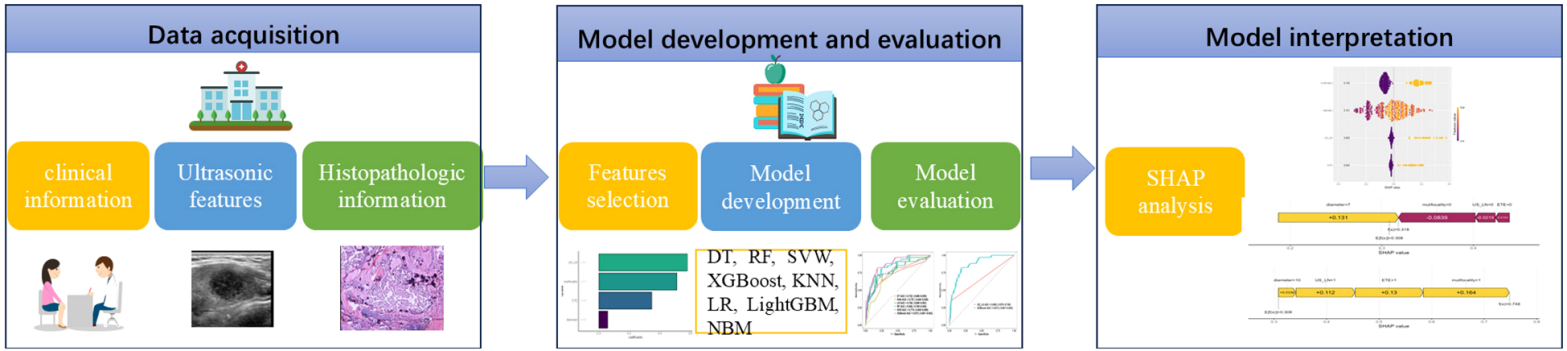
Artificial intelligence workflow and study flowchart. DT, decision tree; RF, random forest; SVM, support vector machine; XGBoost, Extreme Gradient Boosting; KNN, k-nearest neighbors; LR, logistic regression; LightGBM, light gradient boosting machine; NBM, naive bayes model; SHAP, Shapley Additive Explanation.

Inclusion Criteria: ① Postoperative pathology confirmed the presence of PTC; ② Lesion sizes were less than 10 mm in the greatest dimension. Exclusion criteria: ① A prior history of thyroid surgery; ② Incomplete postoperative pathological results of CLNM; ③ Absence of ultrasound results and thyroid function tests conducted within one month prior to surgery; ④ Patients with other primary malignant tumors. A total of 710 PTMC patients were screened from 2450 patients for model development and randomly split into a training set (496 patients) and a validation set (214 patients) in a 7:3 ratio (**Figure 1**). The analytical workflow is illustrated in **Figure 2**.

### Data acquisition

The clinical, laboratory, and preoperative ultrasound characteristics of patients with PTMC were retrospectively analyzed. The primary clinical indicators for this study encompassed age, gender, and the presence of Hashimoto’s thyroiditis. The laboratory parameters evaluated included triiodothyronine (T3), tetraiodothyronine (T4), free triiodothyronine (FT3), free tetraiodothyronine (FT4), thyroid-stimulating hormone (TSH), thyroglobulin antibody (TGAb), thyroid peroxidase antibody (TPOAb), and thyrotrophin receptor antibody (TRAb). According to the American College of Radiology Thyroid Imaging, Reporting and Data System(ACR TI-RADS) (14), our study focused on key ultrasound features: multifocality, tumor diameter, lesion location (isthmus, upper, middle, lower), composition (solid, spongiform, mixed, cystic), echogenicity (very hypoechoic, hypoechoic, isoechoic, anechoic), margin (smooth, ill-defined, irregular, ETE), shape (wider-than-tall, taller-than-wider), echogenic foci (none, large comet-tail artifacts, punctate foci, peripheral calcification, macrocalcification), and cervical lymph nodes status based on ultrasound (US-LN). For cases involving multifocal lesions, we documented the ultrasonographic characteristics of the lesion exhibiting the highest ACR TI-RADS classification.

Tumor diameter was defined as the maximum diameter in long axial section. ETE was defined as gross extrathyroidal extension, suspicious minor ETE, and capsule contact. Microcalcification is defined as calcification with a diameter ≤1 mm. Two experienced sonographers, each with over 10 years in thyroid ultrasound, evaluated the images. In case of differing opinions, they discussed to reach a final decision.

### Feature selection

A comprehensive set of 21 variables encompassing clinical characteristics, thyroid function parameters, and ultrasound features were meticulously selected for analysis. Feature screening is an important part of model construction. To pick out a representative set of composite features, we used a two-stage feature-selection procedure (15). First, Support Vector Machine-Recursive Feature Elimination (SVM-RFE) with fivefold cross-validation and the Least Absolute Shrinkage and Selection Operator (LASSO) were employed as preliminary feature selection methods (16, 17). Subsequently, features that received the majority of votes from both methods were included in the optimal feature set. SVM-RFE is a ML methodology that employs SVM to discern the most pertinent variables through an iterative process of feature elimination from the feature vector produced by the SVM algorithm (18). LASSO regression identifies pertinent variables by optimizing the parameter λ to minimize classification error (19). The features of SVM-RFE and LASSO regression were analyzed using multivariate logistic regression, leading to the identification of the most significant features. This technique is primarily employed for feature selection and the construction of an optimal classification model.

### Model development and evaluation

Using the optimal feature set, eight ML algorithms were employed to construct models based on the training data set. The algorithms encompassed in this study include (https://scikit-learn.org/stable/):

1. Decision Tree (DT): A rule-based model that splits data hierarchically using feature thresholds to make predictions.
2. Random Forest (RF): An ensemble of DTs trained with bootstrapped data and random feature subsets, aggregating outputs to reduce overfitting.
3. k-Nearest Neighbors (KNN): A lazy learner that classifies instances based on majority votes or averages from the k closest training samples.
4. Support Vector Machine (SVM): A margin-maximizing classifier that separates classes using hyperplanes, aided by kernels for non-linear data.
5. Extreme Gradient Boosting (XGBoost): A gradient-boosted DT framework optimizing loss functions with regularization and sequential error correction.
6. Naive Bayes Model (NBM): A probabilistic classifier assuming feature independence, applying Bayes’ theorem for likelihood estimation.
7. Logistic Regression (LR): A linear model predicting class probabilities via sigmoid-transformed weighted feature sums.
8. Light Gradient Boosting Machine (LightGBM): A high-efficiency boosting algorithm growing tree leaf-wise with histogram-based speed optimizations.

Subsequently, the performance of each ML model was assessed utilizing the internal validation set. Postoperative pathological results served as the gold standard for comparison. Metrics such as area under the curve (AUC), sensitivity, specificity, recall rate, accuracy, and F1 scores were employed to evaluate and compare the performance of the ML models. The model demonstrating the highest AUC was determined to be the optimal model. The performance of this optimal ML model was compared to the efficacy of ultrasound in assessing lymph node metastasis. Furthermore, the net benefit in clinical utility of the ML models was evaluated in the validation set using decision curve analysis (DCA).

### Interpretable ML models

To deepen our comprehension of the individual contributions of features to the classification process, we apply the SHAP algorithm. This algorithm leverages a game-theoretic framework to elucidate the outputs of ML models, thereby enabling a rigorous evaluation of feature importance within these methodologies (20).

### Statistical analysis

Data normality was assessed with the Kolmogorov-Smirnov test. Data conforming to a normal distribution were expressed as mean ± standard deviation, and comparisons between two groups were conducted using the independent t-test. For data that did not conform to a normal distribution, the median and interquartile range (M [Q1, Q3]) were employed. Comparisons between groups were performed using the Mann-Whitney U test. Categorical variables were represented as percentages (%), and intergroup comparisons were conducted utilizing Pearson’s chi-square test or Fisher’s exact test. The DeLong test was employed to compare the AUC across various models. The statistical analyses and modeling processes were performed using the R software package (version 4.2.1) and DCPM (version 4.01, Jingding Medical Technology Co., Ltd.). A two-sided P-value of less than 0.05 was considered to indicate statistical significance.

## Results

### Baseline characteristics

A total of 710 patients (Median age (IQR), 43.0 years (35.0-54.8); 198 men) diagnosed with PTMC were included in this study, with 496 patients (70%) allocated to the training set and 214 patients (30%) designated as the internal validation set (**Figure 1**, **Table 1**). The comprehensive baseline clinical characteristics of the training and validation cohorts are presented in **Table 1**. No significant differences were observed in clinical features between the two groups (all P > 0.05).

**Table 1:** Baseline characteristics of PTMC patients in the training and validation sets.

Characteristic	ALL (n=710)	Training set (n=496)	Validation set (n=214)	*P*-value
Age, Median (IQR)	43.0 (35.0-54.8)	43.0 (35.0-55.0)	42.5 (33.0-53.0)	0.217
Gender, n (%)				0.607
Female	512 (72.1)	361 (72.8)	151 (70.6)	
Male	198 (27.9)	135 (27.2)	63 (29.4)	
Hashimoto’s thyroiditis, n (%)				
No	530(74.7)	369(67.7)	161(75.2)	0.887
Yes	180(25.4)	127(25.6)	53(24.8)	
Multifocality, n (%)				0.318
No	472 (66.5)	336 (67.7)	136 (63.6)	
Yes	238(33.5)	160(32.3)	78 (36.5)	
Diameter (mm), median (IQR)	6.9 (5.1-8.7)	6.9 (5.1-8.7)	6.9 (5.3-8.6)	0.644
Extrathyroidal extension, n (%)				0.853
No	576 (81.1)	401 (80.9)	175 (81.8)	
Yes	134 (18.9)	95(19.2)	39 (18.2)	
Thyroid function, median (IQR)				
T4(ug/dl)	7.2 (6.3-8.1)	7.2 (6.3-8.1)	7.4 (6.3-8.1)	0.402
T3(ng/dl)	107.7(94.3-119.0)	107.5(94.2-118.7)	108.9(95.4-119.9)	0.266
TSH (uIU/ml)	1.9 (1.3-2.6)	1.8 (1.3-2.6)	1.9 (1.4-2.5)	0.561
FT3(pg/ml)	2.9 (2.6-3.2)	2.9 (2.6-3.1)	2.9 (2.7-3.2)	0.052
FT4(ng/dl)	1.2 (1.1-1.4)	1.2 (1.1-1.4)	1.2 (1.1-1.4)	0.221
TPOAb (IU/ml)	11.5 (8.1-17.5)	11.6 (8.1-18.5)	10.8 (8.1-16.4)	0.522
TGAb (IU/ml)	15.4 (12.8-34.1)	15.4 (12.8-34.1)	15.3 (13.0-33.9)	0.661
TRAb (IU/L)	0.80 (0.67-1.14)	0.80 (0.67-1.13)	0.81 (0.67-1.17)	0.739

IQR, Interquartile range; T4, tetraiodothyronine; T3, triiodothyronine; TSH, thyroid-stimulating hormone; FT3, free triiodothyronine; FT4, free tetraiodothyronine; TPOAb, thyroid peroxidase antibody; TGAb, thyroglobulin antibody; TRAb, thyrotrophin receptor antibody.

Out of 710 patients in the study, 234 (32.95%) were confirmed to have CLNM. This study revealed that patients with CLNM metastasis were significantly younger than those without metastasis (P = 0.03) and presented with larger lesion diameters relative to their non-metastatic counterparts (P < 0.001). Furthermore, the prevalence of multifocal lesions and ETE was significantly higher in male patients with metastasis (P < 0.05). In the training set of 496 patients, 162 individuals (32.66%) developed CLNM. Within this subgroup, the proportions of male patients, multifocal lesions, tumor diameter, ETE, and abnormal US-LN were higher compared to patients without CLNM. Similarly, in the validation set of 214 patients, 72 individuals (33.64%) developed CLNM. Among these patients, the proportions of multifocal lesions, tumor diameter, and US-LN were elevated relative to those without CLNM. A comparative analysis of clinical, laboratory (**Table 2**), and ultrasonic characteristics (**Table 3**) between CLNM-positive and CLNM-negative patients in the training and validation sets. Our findings indicate that male patients, presence of ETE, and suspicious US-LN are linked to a higher risk of CLNM. Furthermore, a positive correlation has been observed between lesion diameter and the incidence of CLNM. Conversely, the presence of Hashimoto’s thyroiditis appears to be negatively correlated with the occurrence of CLNM.

**Table 2:** Baseline characteristics of patients in the training and testing datasets.

	Training set			Validation set		
Characteristics	CLNM (-) (n=334)	CLNM (+) (n=162)	*P*-value	CLNM (-) (n=142)	CLNM (+) (n=72)	*P*-value
Age, Median (IQR)	44.0(36.0-55.0)	42.0(34.0-54.8)	0.127	44.0(34.3-54.8)	38.0 (32.8-51.3)	0.115
Gender, n (%)			0.043			0.465
Female	253 (75.8)	108 (66.7)		103 (72.5)	48 (66.7)	
Male	81 (24.3)	54 (33.3)		39 (27.5)	24 (33.3)	
Hashimoto’s thyroiditis, n (%)			0.031			0.577
No	241 (72.2)	128 (79.0)		103 (72.5)	58 (80.6)	
Yes	93 (27.8)	34 (21.0)		39 (27.5)	14 (19.4)	
Laboratory test, Median (IQR)						
RBC, 10^9^/L	4.3(4.1-4.5)	4.4(4.0-4.8)	0.011	4.3(4.1-4.5)	4.4(4.0-4.8)	0.276
PLT, 10^9^/L	116(81-128)	119(83-156)	0.025	103(80-129)	117(89-143)	0.215
Thyroid function, Median (IQR)						
T4, (ug/dl)	7.2 (6.2-8.0)	7.3(6.4-8.2)	0.588	7.4 (1.4)	7.1 (1.3)	0.126
T3, (ng/dl)	107.0(95.7-116.7)	108.1(92.8-120.8)	0.619	107.6(95.8-119.9)	111.3 (94.3-119.3)	0.686
TSH, (uIU/ml)	1.8 (1.3-2.6)	1.9 (1.4-2.6)	0.239	2.0 (1.4-2.6)	1.8 (1.4-2.4)	0.378
FT3, (pg/ml)	2.9 (2.6-3.1)	2.9 (2.6-3.2)	0.155	2.9 (2.7-3.2)	2.9 (2.7-3.2)	0.916
FT4, (ng/dl)	1.2 (1.1-1.4)	1.2 (1.1-1.4)	0.906	1.3 (1.1-1.4)	1.2 (1.2-1.4)	0.491
TPOAb, (IU/ml)	12.0 (8.2-18.6)	10.6 (8.1-17.5)	0.236	11.6 (8.3-15.9)	10.2 (8.1-17.1)	0.333
TGAb, (IU/ml)	15.5 (13.0-32.6)	14.7(12.3-43.6)	0.521	15.1(13.2-39.3)	15.4(13.0-21.0)	0.997
TRAb, (IU/L)	0.8 (0.7-1.1)	0.8 (0.7-1.2)	0.703	0.9 (0.7-1.2)	0.8 (0.7-1.0)	0.169

CLNM, central lymph node metastasis; IQR, Interquartile range; RBC, red blood cell; PLT: platelet; T4, tetraiodothyronine; T3, triiodothyronine; TSH, thyroid-stimulating hormone; FT3, free triiodothyronine; FT4, free tetraiodothyronine; TPOAb, thyroid peroxidase antibody; TGAb, thyroglobulin antibody; TRAb, thyrotrophin receptor antibody.

**Table 3:** Ultrasonic characters of the patients with papillary thyroid microcarcinoma.

	Training set			Validation set		
Characteristics	CLNM (-) (n=334)	CLNM (+) (n=162)	*P*-value	CLNM(-) (n=142)	CLNM (+) (n=72)	*P*-value
Median diameter (IQR), (mm)	6.2(4.6-8.5)	7.8(6.5-9.2)	<0.001	6.8(4.9-8.5)	7.4(6.4-8.8)	0.016
Tumor number, n (%)			<0.001			<0.001
Single	257 (77.0)	79 (48.8)		105 (73.9)	31 (43.1)	
Multiple	77 (23.1)	83 (51.2)		37 (26.1)	41 (56.9)	
Location, n (%)			0.759			0.166
Isthmus	22 (6.6)	11 (6.8)		8 (5.63)	9 (12.50)	
Upper portion	79 (23.7)	43 (26.5)		29 (20.4)	19 (26.4)	
Middle portion	97 (29.0)	50 (30.9)		39 (27.5)	14 (19.4)	
Lower portion	136 (40.7)	58 (35.8)		66 (46.5)	30 (41.7)	
Composition, n (%)			0.097			1.000
Nonsolid	7 (2.1)	8 (4.9)		3 (2.1)	2 (2.8)	
Solid	327 (97.9)	154 (95.1)		139 (97.9)	70 (97.2)	
Echogenicity, n (%)			0.720			0.178
Isoechoic	8 (2.4)	2 (1.2)		2 (1.4)	4 (5.6)	
Very hypoechoic	163 (48.8)	75 (46.3)		75 (52.8)	31 (43.1)	
Hyperechoic	1 (0.3)	0 (0.0)		1 (0.7)	0 (0.0)	
Hypoechoic	162 (48.5)	85 (52.5)		64 (45.1)	37 (51.4)	
Margin, n (%)			0.184			0.615
Smooth and ill-defined	135(40.4)	75(46.3)		65(45.8)	37 (51.4)	
Irregular	199 (59.6)	87 (53.7)		77 (54.2)	35 (48.6)	
Extrathyroidal extension, n (%)			<0.001			0.205
No	294 (88.0)	107 (66.1)		120 (84.5)	55 (76.4)	
Yes	40 (12.0)	55 (34.0)		22 (15.5)	17 (23.6)	
Shape, n (%)			0.993			1.000
Wider-than-tall	146 (43.7)	70 (43.2)		64 (45.1)	32 (44.4)	
Taller-than-wider	188 (56.3)	92 (56.8)		78 (54.9)	40 (55.6)	
Calcification, n (%)			0.109			0.430
No calcification	157 (47.0)	60 (37.0)		67 (47.2)	28 (38.9)	
Microcalcification	145 (43.4)	83 (51.2)		57 (40.1)	37 (51.4)	
Peripheral calcification	1 (0.3)	2 (1.2)		1 (0.7)	0 (0.0)	
Macrocalcification	31 (9.3)	17 (10.5)		17 (12.0)	7 (9.7)	
US Lymph nodes, n (%)			<0.001			<0.001
Nonsuspicious	305 (91.3)	112 (69.1)		133 (93.7)	48 (66.7)	
Suspicious	29 (8.7)	50 (30.9)		9 (6.3)	24 (33.3)	

CLNM, central lymph node metastasis; IQR, interquartile range; US, ultrasound.

### Feature selection

**Figure 3:**
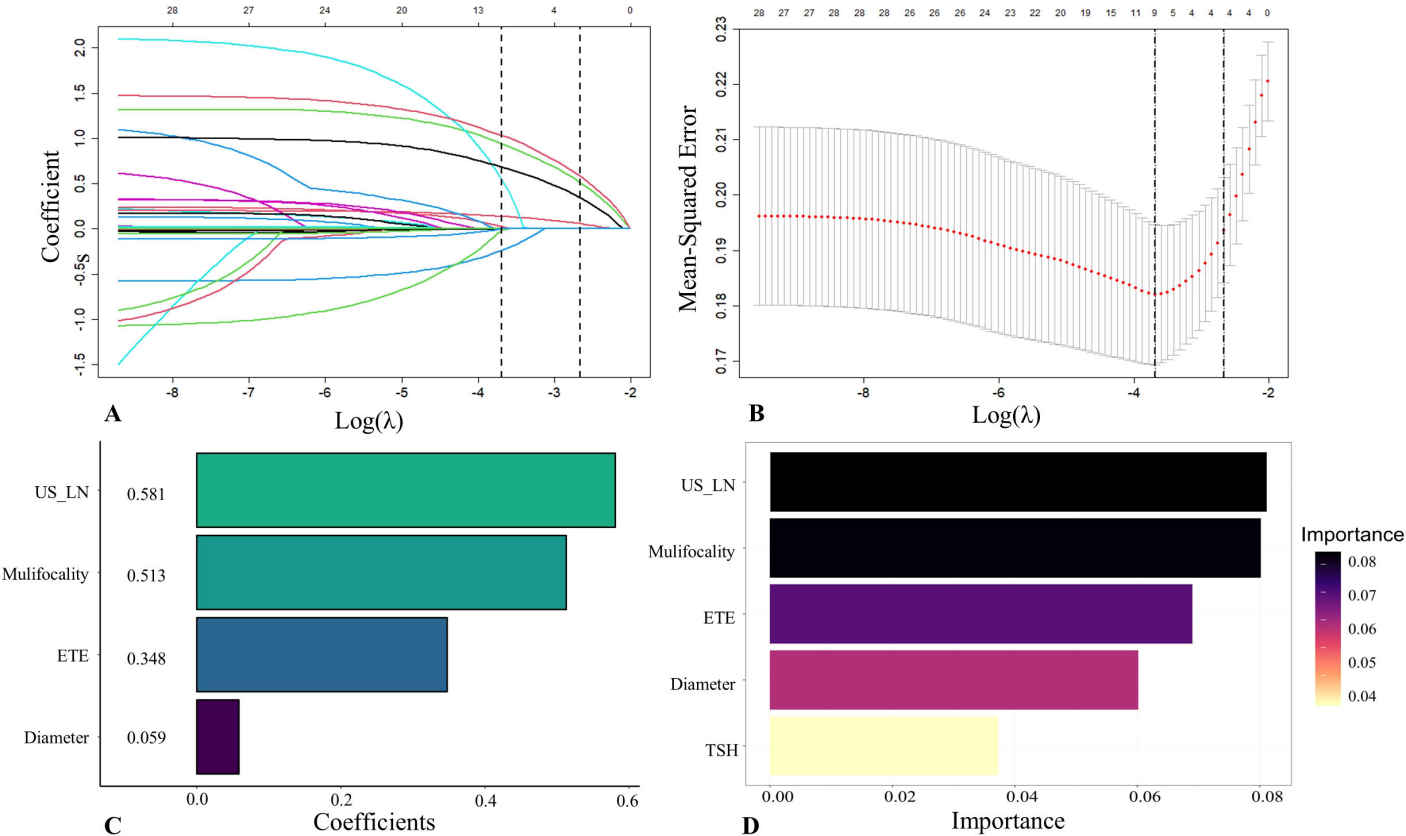
Feature selection was performed by the Least Absolute Shrinkage and Selection Operator (LASSO) regression and Support Vector Machine Recursive Feature Elimination (SVM-RFE). **(A)** Coefficients derived from LASSO regression. **(B)** The range of optimal values was identified by the LASSO model. **(C)** Four optimal features were chosen by the LASSO. **(D)** Five optimal features were chosen by the SVW-RFE. US-LN, cervical lymph nodes status based on ultrasound; ETE, extrathyroidal extension; TSH, thyroid-stimulating hormone.

The 21 recruited features, encompassing clinical characteristics, thyroid function metrics, and ultrasound attributes, underwent feature selection utilizing LASSO regression in conjunction with the SVM-RFE algorithm. The criterion used is one standard error from the minimum mean squared error (lambda.1se). The LASSO method chose the penalty parameter λ at 0.069, based on one standard error from the minimal mean squared error, yielding 4 non-zero coefficients. Dotted vertical lines mark the optimal values in **Figure 3A**. The LASSO regression identified four non-zero coefficient features: US-LN, multifocality, ETE, and diameter, as shown in **Figures 3A-C**. The feature selection outcomes based on the SVW-RFE algorithm were depicted in **Figure 3D**. Here, the top five variables, ranked by parameter importance, were US-LN, multifocality, ETE, diameter, and TSH. The multivariate logistic regression analysis revealed that the presence of US-LN (odds ratio [OR] = 3.77, P < 0.001), ETE (OR = 2.47, P = 0.001), multifocality (OR = 3.27, P< 0.001), and tumor diameter (OR = 1.22, P < 0.001) were significant predictors of CLNM. In contrast, TSH was not found to be significant (OR=1.011, P= 0.609) (**Table 4**). Finally, the optimal features we selected were US-LN, multifocality, ETE, and diameter.

**Table 4:** Multivariate logistic regression results of predictors of cervical lymph node metastasis.

Characteristics	B	SE	OR	95%CI	*Z*-value	*P*-value
US_LN	1.327	0.27922	3.77	3.769 (2.191-6.569)	4.752	0
ETE	0.905	0.26432	2.471	2.471 (1.472-4.159)	3.423	0.001
Multifocality	1.186	0.22103	3.275	3.274 (2.128-5.068)	5.367	0
diameter	0.197	0.05292	1.218	1.217 (1.098-1.352)	3.72	0
TSH	0.011	0.02191	1.011	1.011 (0.970-1.065)	0.511	0.609

US-LN, cervical lymph nodes status based on ultrasound; ETE, Extrathyroidal extension; TSH, thyroid-stimulating hormone; B, Coefficient of regression; SE, Standard error; OR, odds ratios; CI, confidence intervals.

### Model performance comparison and clinical practicality

**Figure 4:**
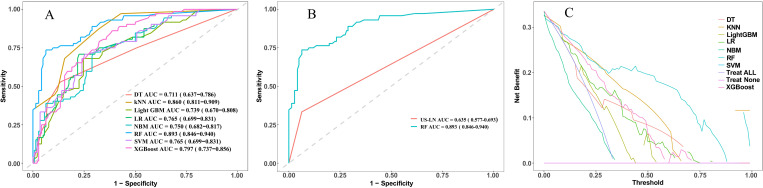
Presents a comparative analysis of various machine learning models employed for the prediction of cervical lymph node metastasis (CLNM) in papillary thyroid microcarcinoma (PTMC) patients. **(A)** ROC curves of the eight model in the validation set. **(B)** ROC curves evaluate the RF model and US-LN through AUC scores. **(C)** Decision curve analysis in the validation set. ROC, receiver operating characteristic; AUC, area under the ROC curve; DT, decision tree; KNN, k-nearest neighbors; LightGBM, light gradient boosting machine; LR, logistic regression; NBM, naive bayes model; RF, random forest; SVW, support vector machine; XGBoost, Extreme Gradient Boosting.

**Figure 5:**
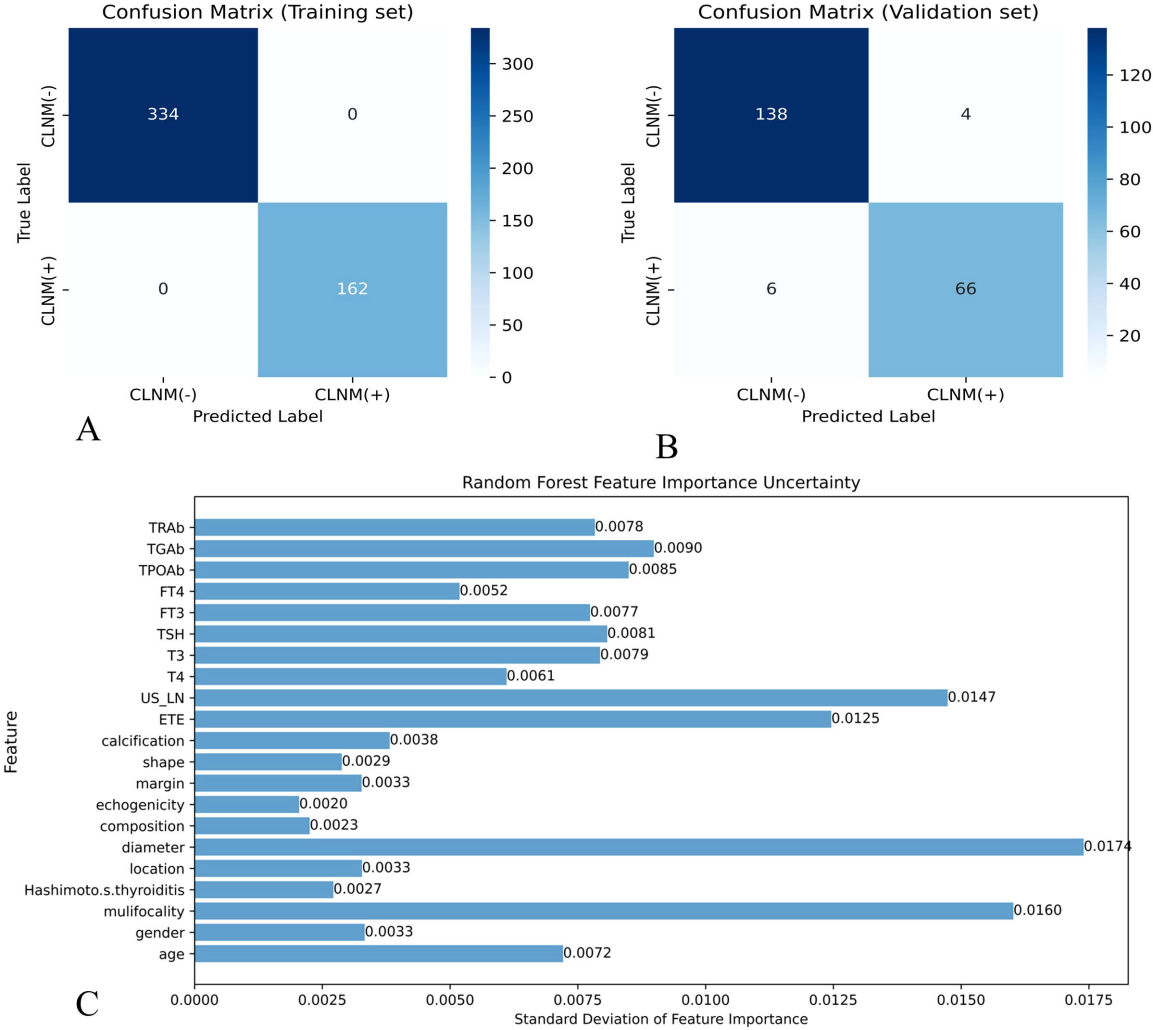
Effectiveness evaluation of RF prediction models **(A)** Confusion matrix of RF in the training set. **(B)** Confusion matrix of the RF model in the validation set. X-axis represents the model prediction, y-axis represents the real situation, and the values in the box are the number of samples. **(C)** Standard deviation of feature importance in the RF model.

In this study, eight ML models were utilized to develop a predictive model for CLNM in patients with PTMC. The predictive model incorporated variables including US-LN, multifocality, ETE, and tumor diameter. **Table 5** illustrates the predictive performance of eight models in forecasting CLNM in patients with PTMC for both the training and validation sets. Notably, the RF model exhibits superior performance in both datasets. As shown in **Figure 4A**, the validation set showed that the RF model had the best predictive performance for CLNM in PTMC patients (AUC = 0.893), followed by KNN (AUC = 0.860), XGBoost (AUC = 0.797), LR (AUC = 0.765), SVM (AUC = 0.765), NBM (AUC = 0.750), LightGBM (AUC = 0.739), and DT (AUC = 0.711). Notably, the US-LN achieved an AUC of 0.635 (95% CI: 0.577-0.693) in the validation set (**Figure 4B**). The Delong test indicated that the diagnostic efficiency of the RF model was significantly superior to that of the other seven models and the US-LN, with a statistically significant difference (P < 0.05). The RF model demonstrated better performance metrics, with accuracy at 0.832, specificity at 0.866, positive predictive value at 0.743, and F1-score at 0.753. The RF model exhibited robust discriminative performance on the training set (n=496), accurately classifying 334 out of 496 cases (67.3%) as CLNM-negative (true negatives) and 162 out of 496 cases (32.7%) as CLNM-positive (true positives). Analysis of the confusion matrix indicated a balanced error distribution, with no discernible systematic bias toward either class (refer to **Figures 4A, B**). **Figures 5A–C** shows that uncertainty in the RF model coefficients was assessed using 1,000 Bootstrap iterations. The standard deviation (SD) of feature importance scores indicated strong stability for the top predictors (US-LN: 0.0147; diameter: 0.0174; multifocality: 0.0160; ETE: 0.0125), while TSH showed more variability (SD = 0.12), warranting cautious interpretation. This analysis confirms the reliability of the key clinical and imaging features in predicting CLNM. To evaluate the models’ clinical benefit, we used DCA to plot net benefit against risk threshold. The pastel purple dashed line represents the projected net benefit associated with ‘no intervention,’ whereas the solid purple dashed line illustrates the anticipated net benefit corresponding to ‘full intervention’. Given that threshold probabilities differ among patients, the net benefit is assessed across a spectrum of probabilities. Decision curve analysis (**Figure 4C**) revealed the RF model provided the highest net benefit across the clinically relevant threshold probability range of 30-80%, outperforming both extreme strategies (‘treat all’ and ‘treat none’) and all other ML models. This supports its utility for preoperative CLNM risk assessment in PTMC patients.

**Table 5:** Diagnostic efficacies of seven machine learning methods and US-LN.

Classifier	AUC (95%CI)	*P*-value	Accuracy	Sensitivity	Specificity	PPV	NPV	F1-Score
Training set								
DT	0.760(0.715~0.805)	<0.001	0.826	0.734	0.863	0.681	0.890	0.734
RF	0.957(0.941~0.973)	–	0.887	0.858	0.901	0.808	0.929	0.832
XGBoost	0.854(0.821~0.887)	<0.001	0.734	0.914	0.647	0.556	0.939	0.692
SVW	0.770(0.727~0.813)	<0.001	0.268	0.327	0.240	0.173	0.423	0.226
KNN	0.920(0.896~0.943)	0.001	0.821	0.907	0.778	0.665	0.946	0.768
LR	0.777(0.735~0.820)	<0.001	0.759	0.652	0.807	0.600	0.839	0.625
LightGBM	0.765(0.722~0.808)	<0.001	0.746	0.636	0.799	0.606	0.819	0.621
NBM	0.767(0.724~0.810)	<0.001	0.710	0.735	0.698	0.541	0.844	0.623
US_LN	0.611(0.572~0.650)	<0.001	0.716	0.633	0.731	0.309	0.913	0.415
Validation set								
DT	0.711(0.637~0.786)	<0.001	0.740	0.667	0.768	0.529	0.855	0.590
RF	0.893(0.846~0.940)	–	0.832	0.764	0.866	0.743	0.879	0.753
XGBoost	0.797(0.737~0.856)	<0.001	0.682	0.889	0.578	0.516	0.911	0.653
SVW	0.765(0.699~0.831)	<0.001	0.266	0.319	0.239	0.176	0.410	0.227
KNN	0.860(0.811~0.909)	0.121	0.743	0.875	0.676	0.578	0.914	0.696
LR	0.765(0.699~0.831)	0.002	0.771	0.647	0.839	0.688	0.813	0.667
LightGBM	0.739(0.670~0.808)	<0.001	0.729	0.681	0.754	0.583	0.823	0.628
NBM	0.750(0.682~0.817)	<0.001	0.692	0.736	0.669	0.530	0.833	0.616
US_LN	0.635(0.577~0.693)	<0.001	0.734	0.727	0.735	0.333	0.937	0.457

US-LN, cervical lymph nodes status based on ultrasound; AUC, area under the receiver operating characteristic curve; PPV, positive predictive value; NPV, negative predictive value; DT, decision tree; RF, random forest; XGBoost, Extreme Gradient Boosting; SVW, support vector machine; KNN, k-nearest neighbors; LR, logistic regression; LightGBM, light gradient boosting machine; NBM, naive bayes model.

### Model interpretability

**Figure 6:**
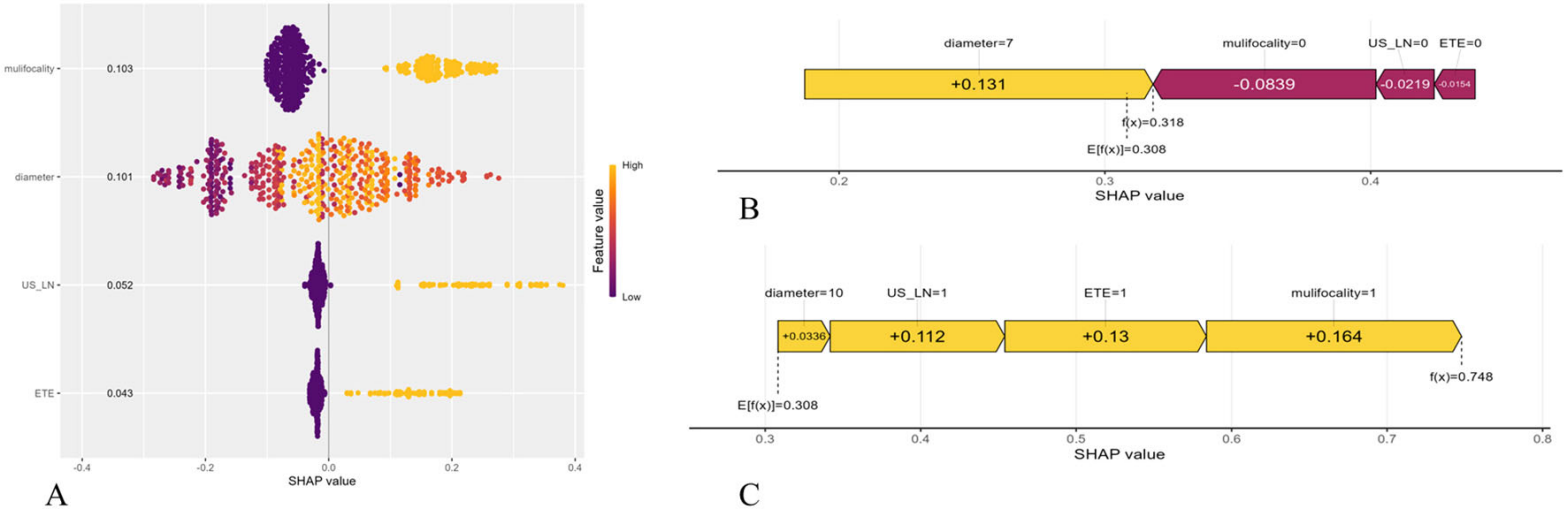
Shapley Additive Explanation (SHAP) of the model. **(A)** Summary plots for the validation sets with associated SHAP values. Each point represents a SHAP value for a patient’s characteristic. **(B)** SHAP force plot for a PTMC patient without CLMN. **(C)** SHAP force plot for a PTMC patient with CLMN. US-LN, cervical lymph nodes status based on ultrasound; ETE, extrathyroidal extension.

We employed the SHAP to enhance the interpretability of the RF model. The feature importance ranking, as illustrated in **Figure 6A**, revealed that multifocality (mean absolute SHAP value = 0.103), tumor diameter (0.101), US-LN (0.052), and ETE (0.043) were the four most significant contributors to the prediction of CLNM. These findings align with the variables identified through LASSO and SVM-RFE selection methods. The directional impact of these features is further illustrated in SHAP force plots. In **Figure 6B**, a CLNM-positive patient’s prediction is driven by multifocality (yellow bar), larger tumor diameter, suspicious US-LN, and ETE, all pushing the model output above the base value (f(x) > 0). Conversely, **Figure 6C** shows a CLNM-negative case where the absence of these features (purple bars) reduces the risk score (f(x) < 0). The RF model’s capability to quantify the contributions of individual features significantly enhances its applicability for personalized risk stratification. Furthermore, the visual clarity provided by SHAP plots facilitates clinicians’ comprehension of model decisions, eliminating the necessity for expertise in ML.

## Discussion

In this retrospective study, we identified US-LN, multifocality, ETE, and tumor diameter as preoperative predictors of CLNM in PTMC patients. We developed and validated eight ML models with four parameters, evaluating their predictive performance and clinical utility using the receiver operating characteristic (ROC) curves and DCA curves, followed by a comparative analysis of their performance. We verified the performance of our newly developed the interpretable RF model for predicting CLNM in PTMC patients, which achieved a higher AUC of 0.893 (95% CI:0.846, 0.940) than other ML models, consistent with previous research (21, 22). The RF algorithm excels in resisting overfitting, handling both continuous and categorical data, estimating error rates, and ranking variable importance. Additionally, we use SHAP scores for visual model interpretation to distinguish between CLMN and non-CLMN patients, allowing for personalized risk assessments and detailed insights into individual predictions.

This study found that ultrasound detected CLNM in 47.9% of PTMC cases confirmed by pathology, with suspicious ultrasound lymph nodes being the key preoperative indicator. Ultrasound shows high specificity for assessing cervical lymph node metastasis in PTMC patients. Some researchers have utilized ultrasound features of cervical lymph nodes to predict N1b PTC metastasis pre-surgery, aiding surgical decisions (23). For example, microcalcification and diameter have been identified as key predictors of lymph node metastasis in PTC patients (21). Generally, most abnormal cervical lymph nodes detected by ultrasound were in the lateral neck region. Ultrasound struggles to detect central cervical lymph nodes behind the trachea and pharynx. However, the central cervical lymph nodes is the most common site for lymph node metastasis from PTC. Our findings indicate that metastatic features in lateral cervical lymph nodes suggest CLNM, highlighting ultrasound’s importance in evaluating lymph node metastasis risk in PTMC patients. Previous studies have pinpointed multifocality, ETE, and tumor size as key risk factors for predicting CLNM in PTMC patients (22, 24, 25). ETE involves invasion into nearby muscles, the trachea, and nerves, especially the recurrent laryngeal nerve. Criteria for evaluating external glandular invasion in PTC include disrupted membrane echo or more than 25% contact between the tumor and the membrane. Many studies indicate that ETE is a major risk factor for CLNM. Compared to single lesion, multifocality in PTC patients raise the risk of local tumor progression and higher CLNM rates (24, 25). However, the impact of multifocality on recurrence is still debated, requiring further large-scale studies for clearer evidence.

Tumor diameter is the main criterion for T staging in thyroid cancer and a known independent risk factor for CLNM. Larger tumor size is linked to higher risk of clinical progression, regardless of other factors. There is a moderate correlation between tumor size and the number and percentage of lymph node metastases (26). A study reported 8,668 cases of PTMC and found that CLNM occurred in 22.9% of patients with tumors under 0.5 cm and in 38.0% of those with tumors between 0.5 cm and 1 cm (27). This indicates that the risk of lymph node metastasis increases with tumor size in PTMCs, supporting the current study’s findings. Nevertheless, tumor volume is likely a better predictor of lymph node metastasis risk than tumor size alone. For patients with multifocal disease, evaluating the total tumor volume—summed from the largest diameters of all tumor foci—may more accurately predict lymph node metastasis risk, offering a clearer picture of tumor burden compared to assessing a single tumor focus (28). Moreover, the anatomical location of the tumor plays a critical role in determining the probability of lymph node metastasis. Specifically, neoplasms located in the upper pole of the thyroid gland demonstrate an increased tendency for metastasis to the lateral cervical lymph nodes (29). Therefore, it is crucial to assess tumor size, volume, and location to evaluate the risk of lymph node metastasis in papillary thyroid carcinoma. While tumor size is an important prognostic factor, tumor volume can provide a more detailed risk assessment in some cases. Including the tumor’s location is also vital for developing a personalized treatment strategy.

The prediction of lymph node metastasis in thyroid cancer has advanced from using solely clinicopathological models to incorporating serological, molecular, and imaging markers, and from traditional algorithms like logistic regression to sophisticated ML models (11, 21, 24, 25, 30). Unfortunately, these methods still have limitations. Pathological features could not be obtained noninvasively before surgery, and traditional radiomics’ high-throughput features are influenced by imaging parameters, limiting their clinical application. Although the RF model constructed in this study demonstrated excellent predictive performance (AUC=0.893), its clinical decision-making value requires careful evaluation considering both false-positive and false-negative results. The model demonstrated a specificity of 97.2% during validation, indicating its potential efficacy in accurately identifying patients who are unlikely to benefit from prophylactic CLND. This could lead to a reduction in unnecessary surgeries by approximately 95%, while only failing to detect 2.8% of true cases of CLNM. This finding is particularly significant in addressing a critical clinical need, as emphasized by the ATA guidelines, which advocate for the avoidance of overtreatment in patients with low-risk papillary thyroid microcarcinoma (PTMC) (7). Although the false negative rate of 8.3% necessitates careful consideration, this performance is superior to that of conventional ultrasound, which typically exhibits false negative rates of 20-30% in the assessment of central lymph nodes (31). The 6 missed CLNM (+) cases in validation all had tumor diameters <5mm and no ETE - characteristics where conservative management may still be appropriate. The comparable error distributions between training and validation sets (Δspecificity <3%, Δsensitivity <5%) confirm the model’s reliability across populations, a notable improvement over previous ML approaches that showed greater performance degradation (30). In the future, it is recommended to adjust decision thresholds according to the surgeon’s risk tolerance and to conduct subgroup analyses for borderline cases, such as tumors measuring 5–7 mm.

The DCA results position our RF model as a clinically useful tool across the decision spectrum: it could prevent unnecessary dissection in low-risk patients (thresholds <30%) while reliably identifying high-risk cases needing intervention (thresholds 50-80%). This balanced performance addresses the core dilemma in PTMC management - avoiding both overtreatment and under-treatment. Future research should focus on the following directions: First, the interpretation of ultrasound features (such as US-LN and ETE) is subjective. Future studies could incorporate AI-assisted ultrasound image analysis (e.g., deep learning segmentation algorithms) to reduce human bias (32). Second, the current model is based on static preoperative data, whereas CLNM risk may change with tumor progression. For example, rapidly growing PTMCs may carry a high metastatic risk even if their initial diameter is small (33). Therefore, developing dynamic risk assessment tools (e.g., incorporating follow-up ultrasound parameters) could further refine clinical decision-making.

In addition to its clinical significance, this study introduces several methodological innovations. First, this study employs two popular feature selection methods to identify common variables, as different methods yield varying results and some only indicate variable importance. This approach aims to select the best variables effectively. Second, this represents the inaugural interpretable ML model designed to predict CLNM in patients with PTMC. Previous ML methods do not provide definitive prediction accuracy for individuals. Nowadays, SHAP value and SHAP force plot offer greater convenience. This study presents interpretability of the ML model and shows accurate prediction for CLMN in PTMC patient. Although our RF model achieved high AUC, clinicians should note that predictions for patients with multifocal lesions (SHAP range: 0.2–0.8) carry higher uncertainty than those with clear ETE (SHAP range: 0.6–0.9).Utilizing our model, clinicians can acquire personalized insights regarding the probability of CLNM prior to surgical intervention.

Our study has limitations, including its retrospective, single-center design, which may limit the generalizability of the findings. The sample from one medical center could lead to variability in model performance when applied to larger, more diverse datasets. Secondly, feature selection reduces overfitting, noise, and random errors but might exclude important variables. Additionally, while the study shows ML could be feasible for CLNM risk stratification, further research is needed to confirm these results.

## Conclusions

This study created and validated a ML model to predict CLNM risk in PTMC patients, providing a useful tool for precise surgical decisions. Future work includes multi-center validation, model optimization, and deployment of a web-based clinical tool.

## Data Availability

The raw data supporting the conclusions of this article will be made available by the authors, without undue reservation.
